# Supplementary material for: Association between household food security and socioeconomic status with paternal smoking: Findings from SEANUTS II Malaysia
Source: PLoS One. 2026 Jan 2;21(1):e0338608. doi: 10.1371/journal.pone.0338608 (PMC12818956; doi:10.1371/journal.pone.0338608)
Supplement: S1 Table — (DOCX) [file pone.0338608.s001.docx]

**Table S1**: Household income among self-employed fathers (n=645)

|  | n | Percentage | Mean | SD |
| --- | --- | --- | --- | --- |
| Parents’ Information |  |  |  |  |
| Income (MYR) |  |  | 4,329 | 4,658 |
| Income Groups (MYR) |  |  |  |  |
| 2,208 and below | 238 | 36.9 |  |  |
| 2,209-4,849 | 191 | 29.6 |  |  |
| 4,850-10,959 | 186 | 28.8 |  |  |
| Above10,959 | 30 | 4.7 |  |  |

MYR represents Malaysia ringgit [USD 1= MYR 4.21 (as at 10^th^ September 2025)]; SD represents standard deviation
