# Supplementary material for: Association between household food security and socioeconomic status with paternal smoking: Findings from SEANUTS II Malaysia
Source: PLoS One. 2026 Jan 2;21(1):e0338608. doi: 10.1371/journal.pone.0338608 (PMC12818956; doi:10.1371/journal.pone.0338608)
Supplement: Attachment — Submitted filename: Response to reviewers.docx [file pone.0338608.s002.docx]

We thank the Editor and reviewers for their constructive feedback, which has greatly helped to improve this manuscript. The authors’ responses are provided in blue font within this document.

**Editor’s Comments**

When submitting your revision, we need you to address these additional requirements.

1. Please ensure that your manuscript meets PLOS ONE's style requirements, including those for file naming. The PLOS ONE style templates can be found at

<https://journals.plos.org/plosone/s/file?id=wjVg/PLOSOne_formatting_sample_main_body.pdf> and 
<https://journals.plos.org/plosone/s/file?id=ba62/PLOSOne_formatting_sample_title_authors_affiliations.pdf>

This revised manuscript has been prepared in full accordance with PLOS ONE’s style requirements

2. Thank you for stating the following financial disclosure:

“This study was conducted as part of the South East Asian Nutrition Surveys II (SEANUTS II) (NN-2018-159) and was funded by FrieslandCampina, Amersfoort, The Netherlands. FrieslandCampina was not involved in the recruitment of children and the final set of results.”

Please state what role the funders took in the study.  If the funders had no role, please state: "The funders had no role in study design, data collection and analysis, decision to publish, or preparation of the manuscript." If this statement is not correct you must amend it as needed.

Please include this amended Role of Funder statement in your cover letter; we will change the online submission form on your behalf.

The role of the funders has been clarified, and the funding statement has been included in the cover letter.

3. Please note that funding information should not appear in the Acknowledgments section or other areas of your manuscript. We will only publish funding information present in the Funding Statement section of the online submission form. Please remove any funding-related text from the manuscript.

The funding statement has been removed from the manuscript.

4. Thank you for stating the following in the Competing Interests section:

“Ilse Khouw is an employee of FrieslandCampina. All other authors declare no competing interest.”

We note that one or more of the authors are employed by a commercial company: FrieslandCampina

1) Please provide an amended Funding Statement declaring this commercial affiliation, as well as a statement regarding the Role of Funders in your study. If the funding organization did not play a role in the study design, data collection and analysis, decision to publish, or preparation of the manuscript and only provided financial support in the form of authors' salaries and/or research materials, please review your statements relating to the author contributions, and ensure you have specifically and accurately indicated the role(s) that these authors had in your study. You can update author roles in the Author Contributions section of the online submission form.

Please also include the following statement within your amended Funding Statement. 
“The funder provided support in the form of salaries for authors [insert relevant initials], but did not have any additional role in the study design, data collection and analysis, decision to publish, or preparation of the manuscript. The specific roles of these authors are articulated in the ‘author contributions’ section.”

If your commercial affiliation did play a role in your study, please state and explain this role within your updated Funding Statement.

The role of the funders has been clarified.

2) Please also provide an updated Competing Interests Statement declaring this commercial affiliation along with any other relevant declarations relating to employment, consultancy, patents, products in development, or marketed products, etc.

Within your Competing Interests Statement, please confirm that this commercial affiliation does not alter your adherence to all PLOS ONE policies on sharing data and materials by including the following statement: ""This does not alter our adherence to  PLOS ONE policies on sharing data and materials.” (as detailed online in our guide for authors <http://journals.plos.org/plosone/s/competing-interests>). If this adherence statement is not accurate and  there are restrictions on sharing of data and/or materials, please state these. Please note that we cannot proceed with consideration of your article until this information has been declared.

Please include both an updated Funding Statement and Competing Interests Statement in your cover letter. We will change the online submission form on your behalf.

The funding statement and competing interest statements have been included in the cover letter.

5. We note that you have indicated that there are restrictions to data sharing for this study. For studies involving human research participant data or other sensitive data, we encourage authors to share de-identified or anonymized data. However, when data cannot be publicly shared for ethical reasons, we allow authors to make their data sets available upon request. For information on unacceptable data access restrictions, please see <http://journals.plos.org/plosone/s/data-availability#loc-unacceptable-data-access-restrictions>.

Before we proceed with your manuscript, please address the following prompts:

a) If there are ethical or legal restrictions on sharing a de-identified data set, please explain them in detail (e.g., data contain potentially identifying or sensitive patient information, data are owned by a third-party organization, etc.) and who has imposed them (e.g., a Research Ethics Committee or Institutional Review Board, etc.). Please also provide contact information for a data access committee, ethics committee, or other institutional body to which data requests may be sent.

b) If there are no restrictions, please upload the minimal anonymized data set necessary to replicate your study findings to a stable, public repository and provide us with the relevant URLs, DOIs, or accession numbers. Please see <http://www.bmj.com/content/340/bmj.c181.long> for guidelines on how to de-identify and prepare clinical data for publication. For a list of recommended repositories, please see <https://journals.plos.org/plosone/s/recommended-repositories>. You also have the option of uploading the data as Supporting Information files, but we would recommend depositing data directly to a data repository if possible.

Please update your Data Availability statement in the submission form accordingly.

The contact information (seanutsmalaysia@ukm.edu.my) for data access has been updated in the statement. This is the official project email registered under the university. Although the email is listed as one of the authors, it represents the entire study group rather than any individual researcher.

6. In this instance it seems there may be acceptable restrictions in place that prevent the public sharing of your minimal data. However, in line with our goal of ensuring long-term data availability to all interested researchers, PLOS’ Data Policy states that authors cannot be the sole named individuals responsible for ensuring data access (<http://journals.plos.org/plosone/s/data-availability#loc-acceptable-data-sharing-methods>).

Data requests to a non-author institutional point of contact, such as a data access or ethics committee, helps guarantee long term stability and availability of data. Providing interested researchers with a durable point of contact ensures data will be accessible even if an author changes email addresses, institutions, or becomes unavailable to answer requests.

Before we proceed with your manuscript, please also provide non-author contact information (phone/email/hyperlink) for a data access committee, ethics committee, or other institutional body to which data requests may be sent. If no institutional body is available to respond to requests for your minimal data, please consider if there any institutional representatives who did not collaborate in the study, and are not listed as authors on the manuscript, who would be able to hold the data and respond to external requests for data access? If so, please provide their contact information (i.e., email address). Please also provide details on how you will ensure persistent or long-term data storage and availability.

The contact information (seanutsmalaysia@ukm.edu.my) for data access has been updated in the statement. This is the official project email registered under the university. Although the email is listed as one of the authors, it represents the entire study group rather than any individual researcher.

7. When completing the data availability statement of the submission form, you indicated that you will make your data available on acceptance. We strongly recommend all authors decide on a data sharing plan before acceptance, as the process can be lengthy and hold up publication timelines. Please note that, though access restrictions are acceptable now, your entire data will need to be made freely accessible if your manuscript is accepted for publication. This policy applies to all data except where public deposition would breach compliance with the protocol approved by your research ethics board. If you are unable to adhere to our open data policy, please kindly revise your statement to explain your reasoning and we will seek the editor's input on an exemption. Please be assured that, once you have provided your new statement, the assessment of your exemption will not hold up the peer review process.

The contact information (seanutsmalaysia@ukm.edu.my) for data access has been updated in the statement. This is the official project email registered under the university. Although the email is listed as one of the authors, it represents the entire study group rather than any individual researcher.

8. One of the noted authors is a group or consortium: SEANUTS II Malaysia Study Group

In addition to naming the author group, please list the individual authors and affiliations within this group in the acknowledgments section of your manuscript. Please also indicate clearly a lead author for this group along with a contact email address.

The lead author of the SEANUTS II Malaysia Study Group is Bee Koon Poh, and a (lead) label has been added after her name.

 9. We note that you have included the phrase “data not shown” in your manuscript. Unfortunately, this does not meet our data sharing requirements. PLOS does not permit references to inaccessible data. We require that authors provide all relevant data within the paper, Supporting Information files, or in an acceptable, public repository. Please add a citation to support this phrase or upload the data that corresponds with these findings to a stable repository (such as Figshare or Dryad) and provide and URLs, DOIs, or accession numbers that may be used to access these data. Or, if the data are not a core part of the research being presented in your study, we ask that you remove the phrase that refers to these data.

We have included the data in Supplementary Table 1.

10. If the reviewer comments include a recommendation to cite specific previously published works, please review and evaluate these publications to determine whether they are relevant and should be cited. There is no requirement to cite these works unless the editor has indicated otherwise.

The reviewers did not specifically request the inclusion of additional citations to particular published works.

**Additional Editor Comments:**

1. Please revise the title to clarify. Does this refer to the association between household food security, socioeconomic status, and paternal smoking? Since this was a cross-sectional study, "association" should be used rather than "relationship."

Thank you for the suggestion. We agree that ‘association’ is the more appropriate term given the cross-sectional design of the study, and the amendment has been made accordingly.

2. The dependent variable of this study is smoking, and the main independent variables are household food security and socioeconomic status. The introduction should summarize an intensive review of the literature on the relationships between these variables and describe the research gaps; however, this is missing. While the discussion section provides information on existing literature, the discussion on what research gaps exist and how this study addresses them remains weak. The study suggests that smoking is prevalent among socioeconomically disadvantaged (food insecure) groups. What new information does this study provide compared to the existing literature? Furthermore, the introduction described conventional cigarette smoking and e-cigarette use, which I expected to assess the relationship between these smoking patterns and household food security and socioeconomic status. However, this analysis was not included, and instead, smoking patterns were presented only descriptively. The revision should focus more on what interesting insights this study offers to international readers compared to existing knowledge.

We thank the reviewer for this constructive comment. The introduction has been revised to provide a more comprehensive review of the literature, highlighting the global and regional burden of smoking and secondhand smoke, as well as the socioeconomic challenges faced by Malaysians. We have also emphasised the limited attention in existing studies to how socioeconomic disadvantage and food insecurity may perpetuate tobacco use within households, with potential harmful implications for children. These revisions strengthen the positioning of the research gap and clarify the unique contribution of our study.

In the discussion, we have strengthened the interpretation of our findings by linking them more explicitly to international evidence while underscoring the novel insights offered. Specifically, this study adds value by:

- **Integrating social science models,** drawing on the Health Belief Model and the Theory of Planned Behaviour to interpret how psychological stress, socioeconomic disadvantage, and family influences shape smoking behaviours. This framing offers international readers a deeper theoretical understanding of stress-smoking pathways in disadvantaged populations.
- Including **policy context and child health implications**. We have linked our findings to local policy implications, including Malaysia’s tobacco control strategies, and highlighted the intergenerational impact of paternal smoking on child nutrition, dietary intake, and health outcomes. These insights broaden the implications for countries facing similar public health and socioeconomic transitions.
- Including some discussion on **contributions beyond existing literature**. By showing that paternal smoking is associated with food insecurity and socioeconomic disadvantage in Malaysia, our study highlights the dual burden of household vulnerability: both economic (reduced food access) and health-related (exposure to secondhand smoke). This perspective offers a new angle for international readers, reinforcing the importance of integrated tobacco control and social protection measures.

**Reviewer 1’s comments**:

Reviewer #1: Congratulations to the author for contributing to a better understanding of this important public health concern—paternal smoking. Below are some suggestions for your consideration. Please take action on any comments you find relevant. Thank you.

Title
• The title is clear and appropriately reflects the study's focus.

Abstract
• [Lines 21-23] The introduction does not clearly articulate the need for the present study. Consider explicitly stating the research gap or rationale.

Thank you for pointing out these issues. We have emphasised the limited attention in existing studies to how socioeconomic disadvantage and food insecurity may perpetuate tobacco use within households, with potentially harmful implications for children. [Lines 24-25]

• [Line 24] There is a slight inconsistency in the recruitment description. While the study recruited children, the analysis focuses on paternal smoking as reported by parents. This could lead to misinterpretation regarding the source of the data. Clarifying this would improve accuracy.

The clarification has been made. Please refer to lines 28-31.

• [Lines 24-26] If the sample is derived from SEANUTS II Malaysia, the sentence should be revised for clarity to ensure accurate representation of the data source.

The clarification has been made. Please refer to lines 26-27.

• [Lines 26-27] It would be beneficial to explicitly mention that information on paternal smoking was gathered through parent/guardian-reported questionnaires.

We added the information about paternal smoking was gathered through parent/guardian-reported questionnaires. [Lines 30-31]

Introduction
• [Overall] Consider adding a transitional sentence to enhance coherence between ideas.

The Introduction has been revised to improve coherence and ensure smoother transitions between ideas.

• [Overall] The introduction highlights the high prevalence of paternal smoking and household food insecurity but does not explicitly state the gap in existing research or the necessity of this study. Strengthening this aspect would improve justification for the research.

Thank you for pointing out these issues. We have emphasised the limited attention in existing studies to how socioeconomic disadvantage and food insecurity may perpetuate tobacco use within households. [Lines 72-76].

Methods
• [Lines 80-82] Instead of solely referring to previous articles, it would be helpful to briefly summarize key aspects of the study design to provide context.

Thanks for the suggestion. The detailed study design has been added in the manuscripts. [Lines 87-92].

• [Line 90] Elaborating on how paternal smoking status was determined—whether based on self-reported frequency, duration, or another criterion—would improve clarity.

The description of paternal smoking has been added. [Lines 114-122]

• [Line 118] "Pearson’s" should be revised for grammatical correctness.

Thank you for pointing out the grammatical errors. The word has been revised.

• [Line 119] Consider using “paternal smoking status” instead of “paternal smoking patterns” to accurately reflect the binary classification in the logistic regression analysis.

The amendment has been made based on the suggestion [Line 135].

• [Line 122] Clarifying the rationale for adjusting Model 2 with “ethnicity” and “area of residence” would strengthen the methodological justification. Why were these particular variables selected for control?

Ethnicity and area of residence were treated as confounders in our analysis. Both variables showed significant associations with smoking status in the univariate model, but became non-significant in the final multivariate model. We retained them in the analysis to account for their potential confounding effects, ensuring that the final estimates of the associations between household food security, socioeconomic status, and paternal smoking were not biased by these variables. This approach is consistent with recommended practices in multivariable modelling, where variables identified as potential confounders are considered even if they do not remain statistically significant in the final adjusted model [Lines 138-141].

Results
• [Line 123] The study does not include socioeconomic status (SES) data for mothers, despite maternal employment, education, and income being key contributors to household SES. Providing a justification for this omission or discussing its potential impact on the findings would enhance the study’s clarity.

This study specifically focused on paternal factors; hence paternal information was the primary interest. We acknowledge that maternal employment and education are also contributors to household SES. To account for overall household circumstances, we included household income, which reflects contributions from both parents. Regarding education, most parents in the sample shared similar education levels, and therefore paternal education was used to represent this variable. For these reasons, only paternal information and household income were included in the analysis.

• [Line 158] What were the selection criteria for variables included in the logistic regression analysis? Clarifying this would enhance the methodological transparency.

We have added the information, please refer to Lines 138-141.

Discussion

• [Lines 175-177] The discussion references associations between dietary intake and children's physiological development. Are these findings within the scope of the current study? If not, consider refining the discussion to align more closely with the study's objectives.

Thank you for the suggestion. We have deleted the sentence, as it does not reflect findings directly captured from our study.

• [Lines 195-197] Since psychological factors were not included as study variables, it would be more appropriate to suggest that psychological distress might be a missing link and recommend further research to explore this relationship. Proposing its inclusion in interventions may be premature without direct evidence from the study, particularly in the Malaysian context.

We thank the reviewer for this observation. The recommendation for further research has been addressed in Lines 241–242.

In addition, a local reference (Rashid et al. 2019) has been included, which provides supporting evidence by demonstrating the relationship between smoking, its determinants, and psychological problems among smokers in an urban Malaysian setting.

• [Lines 187-207] The paragraph structure needs reorganization. Prioritize explaining the study's significant findings and their implications. Recommendations regarding psychological factors and dietary intake should either be placed later in the discussion or moved to the conclusion.

We acknowledge the reviewer’s valuable concern. To maintain the flow and clarity of the discussion, we have placed the statement at the end of the paragraph on food insecurity, as this positioning allows readers to better capture the idea. While psychological factors and dietary intake were not directly examined in this study, highlighting their potential link remains important given that the data were drawn from a children’s survey. This helps underscore possible downstream effects on children’s growth and well-being, and reinforces the need for future studies to explore these relationships in the Malaysian context.

• [Lines 248-251] The recommendation for targeted smoking cessation programs should be incorporated into the conclusion rather than the discussion section.

We thank the reviewer for the suggestion. To improve readability, the recommendations have been summarised and integrated into the conclusion section.

Conclusion
• [Overall] Some recommendations currently presented in the discussion would be more appropriately included here as future directions for addressing smoking prevalence. Consider summarizing key findings and suggesting evidence-based interventions.

The recommendations have been summarised and integrated into the conclusion section.

**Reviewer 2’s comments**:

Reviewer #2: Thank you for inviting me to review the manuscript, title: "Relationship between household food security and socioeconomic status with paternal smoking: Findings from SEANUTS II Malaysia". Manuscript Number: PONE-D-25-11571

 1. Study Design and Methodology

Strengths - The nationwide cross-sectional design and large sample size (n=2,687) enhance generalizability. Ethical approvals and multi-stage cluster sampling are clearly reported.

I want to point out that there are 3 majors issue need to improved in study design and methodology:
i) Missing Definition of Dependent Variable (Paternal Smoking Status) in Method section

While the study provides valuable insights into the association between household food insecurity, socioeconomic status, and paternal smoking in Malaysia, a significant methodological omission is the lack of a clear operational definition for the dependent variable — paternal smoking status — in the Methods section. Although Table 2 distinguishes between conventional cigarette use, e-cigarette use, and dual use, it remains unclear how these categories were collapsed into the binary variable used in the logistic regression model. Specifically, it is not stated whether:

- Only current smokers were included in the "smoker" category,

- Former or occasional smokers were excluded or grouped with non-smokers,

- Any minimum threshold of frequency or duration of smoking was applied.
The binary smoker/non-smoker designation does not account for type (e.g., exclusive vs dual use), intensity (e.g., number of cigarettes/day), duration, or cessation history. Furthermore, data on household smoking behavior (e.g., smoking at home) is described but not utilized in analysis, despite being highly relevant to child exposure. This information is essential for replicability and for interpreting the adjusted odds ratios reported in Table 4. Additionally, the authors note in the Results that "the OR describes the probability of children’s fathers without smoking habits (reference group) relative to those with smoking habits," yet this statement lacks sufficient detail to evaluate the validity of the categorization.

My recommendation:

The Methods section should be revised to include a clear, unambiguous definition of paternal smoking status, including:

- The specific survey question(s) used to assess smoking behavior,

- Criteria used to categorize participants as smokers or non-smokers (e.g., current use of conventional cigarettes, e-cigarettes, or both),

- How occasional, former, or missing responses were handled.

Clarifying this critical aspect of the study design will substantially strengthen the methodological transparency and reproducibility of the manuscript.

Fathers were classified as smokers or vapers if they reported using cigarettes or vapes ‘every day’ or ‘some days’ at the time of the survey, following CDC and WHO surveillance definitions (Kramarow & Elgaddal, 2023). Details on paternal smoking status have been added; please refer to Lines 114–122.

Reference: Kramarow, E.A. and Elgaddal, N., 2023. Current electronic cigarette use among adults aged 18 and over: United States, 2021.

ii) Absence of control for potential psychological variables like stress or mental health in adjusted models / Omitted Confounders

Additionally, while the manuscript extensively discusses psychological stress and coping behavior as drivers of smoking, no mental health variables are included as confounders in the logistic regression models. This raises concern about potential omitted variable bias in the association between socioeconomic status and smoking. The authors should either incorporate these critical variables (if available) or explicitly discuss the limitation.

We appreciate the reviewer’s insightful comment. In our revision, we have highlighted the importance of psychological distress as a potential factor influencing the relationship between food insecurity and smoking. This has been added in the conclusion (Lines 241–242), where we recommend that future studies further investigate its role in the Malaysian context. Additionally, we have acknowledged this issue in the limitations section [Line 329].

iii) No mention of cluster-adjusted standard errors despite multistage cluster sampling

The manuscript describes the use of a multistage cluster sampling strategy to recruit participants, which is appropriate for a nationwide survey. However, no mention is made of whether the statistical analyses accounted for the clustered nature of the data. Ignoring clustering can lead to underestimated standard errors and inflated Type I error rates, potentially compromising the validity of the reported associations. The authors should clarify whether cluster-adjusted standard errors or complex survey procedures were used in the logistic regression models. If not, this limitation should be explicitly acknowledged and discussed.

We appreciate the reviewer’s observation of this important limitation, which we have acknowledged in the limitations section [Lines 330–332].

2. Results and Data Analysis

Strengths - Results are statistically robust, with clear presentation of unadjusted and adjusted logistic regression. Tables are well-structured and easy to interpret.

Critiques & Concerns

a. No multicollinearity check reported for logistic regression model
b. No justification for included covariates in Model 2 (why only ethnicity and area?)
c. Smoking behavior categories (e.g., home smoking, first smoke after waking) are described but not statistically analyzed
d. Lack of reporting on missing data handling strategy

Recommendations
a. Report variance inflation factors (VIF) or other diagnostics to confirm model validity.

b. Provide rationale for covariate selection, or consider including more relevant confounders like child's age.

c. Consider additional analyses (e.g., multinomial regression or stratified models) to explore nuanced behavioral associations.

d. Detail any imputation method used or clarify if complete case analysis was performed.

- Thank you for your comment. We have performed multicollinearity tests and confirmed that no multicollinearity existed among the independent variables. This process has been in the methodology section [Lines 142-145]. We also included two R² values (Cox & Snell and Nagelkerke) to assess the model’s validity, as shown in Table 4
- The process of covariate selection has been added in the methodology section [Lines 138–141]. We also acknowledge that some potential confounders were not considered in the final model, and this has been noted in the limitations.
- Thank you for highlighting this concern. We conducted additional analyses to examine the association of different smoking/vaping types with household food security and SES parameters, and the results are presented below. However, the findings were similar to those in the current Table 4 (where smoking and vaping were grouped together). In addition, the number of vapers or dual users was relatively small (approximately 1:10 compared to non-smokers), which may have affected the statistical significance and reduced the reliability of the results. As shown, the 95% CI widened when the sample was fragmented. Therefore, we decided not to split the groups, and instead used overall smoking behaviours to describe nicotine addiction levels and the potential secondhand smoke exposure of children at home.
- A complete case analysis was conducted [Line 133], and the missing data for smoking behaviours have been indicated in Table 2.

**Table**: Odds ratio for paternal smoking status by household food security and socioeconomic characteristics categories (n=2459)

|  | Vaper (n=117) | | Smoker(n=788) |  | Dual User (n=151) | |
| --- | --- | --- | --- | --- | --- | --- |
|  | **OR** | **95%CI** | **OR** | **95%CI** | **OR** | **95%CI** |
|  |  |  |  |  |  |  |
| Income^a^ (MYR) |  |  |  |  |  |  |
| 2,208 and below | 2.397 | 0.835-6.878 | 3.536*** | 2.202-5.679 | 5.614** | 1.594-19.766 |
| 2,209-4,849 | 3.627** | 1.435-9.171 | 2.622*** | 1.675-4.104 | 5.106** | 1.509-17.277 |
| 4,850-10,959 | 2.222 | 0.921-5.362 | 1.377 | 0.893-2.123 | 3.605* | 1.094-11.874 |
| Above 10,959 | 1 |  | 1 |  | 1 |  |
|  |  |  |  |  |  |  |
| Paternal Education Level^b^ |  |  |  |  |  |  |
| Non-schooling/primary education | 0.316 | 0.041-2.425 | 2.393*** | 1.544-3.710 | 1.528 | 0.611-3.820 |
| Secondary education | 0.836 | 0.529-1.323 | 1.603*** | 1.264-2.032 | 1.390 | 0.902-2.141 |
| Tertiary education | 1 |  | 1 |  | 1 |  |
|  |  |  |  |  |  |  |
| Paternal Occupation^c^ |  |  |  |  |  |  |
| Clerical/ Service and sales staff | 1.013 | 0.590-1.737 | 1.114 | 0.819-1.516 | 1.196 | 0.708-2.022 |
| Agricultural, forestry and fishery/ Craft and trades/ Plant and machine / Elementary workers | 0.439* | 0.206-0.932 | 1.311 | 0.963-1.784 | 1.158 | 0.663-2.023 |
| Armed-forces occupations | 0.432 | 0.054-3.470 | 1.316 | 0.569-3.046 | 1.677 | 0.502-5.597 |
| Self-employed/ Other occupations | 0.630 | 0.346-1.144 | 1.403* | 1.063-1.852 | 0.803 | 0.462-1.398 |
| Not working/ Retiree | 1.126 | 0.242-5.231 | 1.037 | 0.568-1.892 | 0.735 | 0.160-3.372 |
| Professionals/Technicians and associate professionals/Managerial | 1 |  | 1 |  | 1 |  |
|  |  |  |  |  |  |  |
| Paternal Age^d^ (Years) |  |  |  |  |  |  |
| Below 30.0 | 18.837*** | 4.994-71.054 | 1.785* | 1.052-3.028 | 10.814*** | 3.682-31.760 |
| 30.0-39.9 | 6.135** | 1.852-20.330 | 1.315 | 0.951-1.819 | 5.219** | 2.046-13.312 |
| 40.0-49.9 | 1.905 | 0.552-6.570 | 1.054 | 0.764-1.454 | 1.805 | 0.685-4.757 |
| Above 49.9 | 1 |  | 1 |  | 1 |  |
|  |  |  |  |  |  |  |
| Household Food Security^e^ |  |  |  |  |  |  |
| Food insecure | 1.177 | 0.775-1.788 | 1.255* | 1.031-1.528 | 1.461* | 1.015-2.104 |
| Food secure |  |  | 1 |  | 1 |  |

95% CI represents 95% confidence interval; MYR represents Malaysia ringgit [USD 1= MYR 4.21 (as at 10^th^ September 2025)]; OR represent odds ratio.

Significant odds ratio using binary logistic regression at *p<0.05, **p<0.01 and ***p<0.001.

Reference category of paternal smoking is non-smoking.

^a^ Reference category of income group is above MYR10959.

^b^ Reference category of paternal education level is tertiary education level.

^c^ Reference category of paternal occupation is professional/technician/managerial category.

^d^ Reference category of paternal age group is above 49.9 years old.

^e^ Reference category of household food security is food secure; food insecure includes household food insecure, individual food insecure, and child hunger.

3. Writing Quality and Structure

Strengths: Well-written, clear academic language. Structured logically. Abstract summarizes key findings effectively.

Critiques & Concerns : Discussion sometimes reiterates results without deeper interpretation

Recommendations : Emphasize mechanisms, policy implications, and compare more with international findings.

Thank you for the constructive comments that have helped enrich our discussion. We have incorporated several theoretical models, including the Health Belief Model [Lines 278–280] and the Theory of Planned Behaviour [Lines 246–250], into the discussion.

In addition, we have included relevant policies and their implications, such as pictorial warnings on packaging [Lines 280–283] and tax enforcement [Lines 312–313]. Furthermore, in the conclusion, we highlighted proposed policy directions such as the expansion of pro-health taxes, further increases in tobacco taxation, and the gradual phasing out of vaping. These additions strengthen the relevance of our findings to ongoing policy development in Malaysia.

We have also added Southeast Asian context in the introduction [Lines 50–51] and cited references, including Semba (2007).

4. Literature Review and Citations

Strengths: The references are current, relevant, and diverse in origin (including Malaysian and international sources). Well-anchored in public health literature.

Critiques & Concerns

a. Limited discussion of literature from Southeast Asia (other than Malaysia)
b. Some important psychological literature under-cited (stress-smoking pathways)

Recommendations
a. Consider referencing regional studies from Indonesia, Philippines, or Thailand to contextualize findings.
b. Include more theoretical models (e.g., Health Belief Model, Theory of Planned Behavior) to deepen analysis.

Thank you for the constructive comments that have helped enrich our discussion. We have incorporated several theoretical models, including the Health Belief Model [Lines 278–280] and the Theory of Planned Behaviour [Lines 246–250], into the discussion.

In addition, we have included relevant policies and their implications, such as pictorial warnings on packaging [Lines 280–283] and tax enforcement [Lines 312–313]. Furthermore, in the conclusion, we highlighted proposed policy directions such as the expansion of pro-health taxes, further increases in tobacco taxation, and the gradual phasing out of vaping. These additions strengthen the relevance of our findings to ongoing policy development in Malaysia.

We have also added Southeast Asian context in the introduction [Lines 50–51] and cited references, including Semba (2007).

5. Figures and Tables

Strengths: Tables are comprehensive and well-labeled. Statistical details (OR, CI, p-values) are clearly provided.
Critiques & Concerns

a. Table 3: No adjusted percentages for smoking prevalence across SES

b. No figures presented

Recommendations
a. Consider reporting adjusted percentages or use logistic regression margins to show practical effect sizes.
b. Consider including a figure (e.g., forest plot of ORs or a conceptual diagram of model pathways).

We appreciate the reviewer’s observation of this important limitation, which we have acknowledged in the limitations section [Lines 330–332].

Thank you for the suggestion. As the forest plot and Table 4 present the same information, the authors have decided to retain only one to avoid redundancy.

6. Ethical Considerations

Strengths: Ethical approval (UKM JEP-2018-569) and informed consent procedures are clearly documented. Trial registration is provided.

Critiques & Concerns: Data availability is restricted

Recommendations : Consider sharing de-identified or synthetic data or providing an open metadata description.

Thank you for the comment. The data generated or analysed during this study is not publicly available due to confidentiality requirements and compliance with the European General Data Privacy Regulations (GDPR). However, the data is available upon reasonable request via the official project email (seanutsmalaysia@ukm.edu.my), subject to permission from the project funder.

7. Novelty and Significance

Strengths: Novel exploration of paternal smoking in relation to household food insecurity in Malaysia. Addresses a relevant and urgent public health issue.

Critiques & Concerns

a. Causality cannot be inferred (e.g., does food insecurity lead to smoking, or vice versa?)
b. Limited discussion of practical implications beyond general calls for intervention
Recommendations
a. Emphasize this limitation and recommend longitudinal research.
b. Include more specific public health policy suggestions (e.g., integration into tobacco tax reallocation).

Thank you for your suggestion. The limitation and recommendation for adopting a longitudinal design have been included in the revised manuscript [Lines 332–335].

In addition, public health policy suggestions have been described in greater detail in the discussion and summarised in the conclusions.

Most Critical Issues (Prioritized):

- Lack of clear definition of the dependent variable (paternal smoking status) in the Methods section

- Failure to account for multistage cluster sampling in statistical analysis

- Omission of psychological or mental health variables as confounders

- Oversimplification of smoking behavior (e.g., intensity, frequency, cessation)

- Disconnect between stated risks (secondhand/thirdhand smoke) and variables analyzed

- Lack of justification for covariate selection in adjusted regression models

- Missing data and exclusion not properly addressed.

All the concerns raised have been addressed and justified with appropriate reasons. Once again, we sincerely thank you for your constructive feedback and the effort you have taken to help improve our manuscript.

Final Recommendation: Major Revision
