# Supplementary material for: Association between household food security and socioeconomic status with paternal smoking: Findings from SEANUTS II Malaysia
Source: PLoS One. 2026 Jan 2;21(1):e0338608. doi: 10.1371/journal.pone.0338608 (PMC12818956; doi:10.1371/journal.pone.0338608)
Supplement: Attachment — Submitted filename: Response_to_reviewers_auresp_2.docx [file pone.0338608.s003.docx]

We sincerely thank the reviewers for their thorough evaluation and thoughtful comments, which have been invaluable in strengthening our manuscript. Our detailed responses to each point are provided in blue font within this document.

Reviewer #1:

All previous comments have been adequately addressed.

There are a few additional recommendations below, which the journal or authors may consider for further improvement. These suggestions are optional.

[l134–135] It is suggested to remove “household food security levels” and “paternal smoking status” from this section, as the mean and standard deviation values for these variables are not reported in the Results section.

Thank you for highlighting the mistake. We have removed the terms as noted. Please refer to lines 134–135.

[l259–260] The transition towards recommending further study on the association between smoking habits and children’s diet, body weight status, and health appears abrupt and somewhat disconnected from the earlier content in this paragraph, which focused on consequences of household food insecurity. It is recommended to provide additional explanation or linkage to strengthen the rationale for this recommendation.

We sincerely appreciate the reviewers’ careful evaluation of our manuscript and for highlighting the gaps in the discussion. In response, we have revised the relevant sections to better articulate the rationale underlying the association between paternal smoking habits and children’s dietary and health outcomes. The updated content can be found in Lines 259–262.

Reviewer #2:

Overall Assessment

I sincerely appreciate the authors for their thorough and thoughtful revisions. The revised manuscript has significantly improved in clarity, methodological rigor, and interpretability. The authors have addressed all of my major and minor concerns with commendable diligence and transparency.

Response to Previous Comments

Paternal Smoking Status

The authors have successfully incorporated the requested clarifications and adjustments related to paternal smoking status. The revised analytical framework now reflects a more accurate and comprehensive assessment of exposure. This revision strengthens both the internal validity and interpretive depth of the findings.

Psychological Variables (Stress and Mental Health)

The concern regarding the potential influence of psychological variables such as stress or mental health on the outcomes has been thoughtfully acknowledged in the revised limitations section. Although direct measurement of these factors was not feasible, the authors have appropriately discussed their potential confounding effect and justified their absence in the adjusted models.

Cluster-Adjusted Analysis

The issue regarding clustering or potential intra-group correlation has also been addressed. The authors clearly stated the rationale for their analytical approach and acknowledged this aspect as a limitation. Their discussion reflects a sound understanding of the underlying methodological implications.

Minor Revisions and Overall Quality

The manuscript’s language, structure, and presentation have improved markedly. The tables and figures are well-organized, and the discussion section demonstrates greater coherence between empirical findings and theoretical interpretation. The response letter also provides transparent explanations of each revision, which reflects academic rigor and integrity.

Conclusion
In light of the authors’ careful revisions and comprehensive responses to all major and minor comments, I am satisfied that the manuscript now meets the standards required for publication. The study offers valuable insights and will contribute meaningfully to the literature in this area.

Recommendation: Accept for publication.

We would like to take this opportunity to express our sincere gratitude for the reviewers’ agreement with the revisions and the positive feedback on the manuscript.
